# Co-Exposure of Nanopolystyrene and Other Environmental Contaminants—Their Toxic Effects on the Survival and Reproduction of *Enchytraeus crypticus*

**DOI:** 10.3390/toxics10040193

**Published:** 2022-04-15

**Authors:** Luís A. Mendes, Angela Barreto, Joana Santos, Mónica J. B. Amorim, Vera L. Maria

**Affiliations:** 1 Animal Ecology Group (GEA), Universidade de Vigo, 36210 Vigo, Spain; lamendes@ua.pt; 2 Biology Department & CESAM, University of Aveiro, 3810-193 Aveiro, Portugal; abarreto@ua.pt (A.B.); joanasilvasantos@ua.pt (J.S.); mjamorim@ua.pt (M.J.B.A.)

**Keywords:** pharmaceuticals, ecotoxicity, metal-based, plastics, nanomaterials, soil fauna, invertebrates

## Abstract

Plastics in all shapes and sizes have become widespread across ecosystems due to intense anthropogenic use. As such, they can interact with other contaminants that accumulate in the terrestrial environment, such as pharmaceuticals, metals or nanomaterials (NMs). These interactions can potentiate combined toxic effects in the exposed soil organisms, with hazardous long-term consequences to the full ecosystem. In the present study, a terrestrial model species, *Enchytraeus crypticus* (oligochaeta), was exposed through contaminated soil with nanopolystyrene (representative of nanoplastics (NPls)), alone and in combination with diphenhydramine (DPH, representative of pharmaceuticals), silver nitrate (AgNO_3_, representative of metals) and vanadium nanoparticles (VNPs, representative of NMs). AgNO_3_ and VNPs decreased *E. crypticus* reproduction at 50 mg/kg, regardless of the presence of NPls. Moreover, at the same concentration, both single and combined VNP exposures decreased the *E. crypticus* survival. On the other hand, DPH and NPls individually caused no effect on organisms’ survival and reproduction. However, the combination of DPH (10 and 50 mg/kg) with 300 mg NPls/kg induced a decrease in reproduction, showing a relevant interaction between the two contaminants (synergism). Our findings indicate that the NPls can play a role as vectors for other contaminants and can potentiate the effects of pharmaceuticals, such as DPH, even at low and sub-lethal concentrations, highlighting the negative impact of mixtures of contaminants (including NPls) on soil systems.

## 1. Introduction

Industrial development over the past 70 years has brought innovative ideas into daily life, such as plastic-based durable household appliances, as well as nano-based electronics and other applications [1,2]. However, along with the increase in demand, their release into the environment has risen exponentially [3]. One key route for the release and accumulation of these anthropogenic-based contaminants to soil systems is sewage sludge application [4].

Plastic can be degraded into micro (microplastics, MPls) and nano size (nanoplastics, NPls), as a result of mechanical and chemical processes within the soil matrix [5]. Due to the complexity of the matrix and technical limitations in the quantification of NPls, very little is known on their actual distribution [6]. However, similarly to MPls, NPls can interact with the surrounding environment and especially with other existing contaminants, such as pharmaceuticals, metals and/or nanomaterials (NMs) [7,8,9]. Through diverse processes, e.g., aggregation and agglomeration, these contaminants can form complexes with NPls, altering their bioavailability to soil organisms, such as invertebrates and plants [9]. Therefore, there is the concern that the accumulation of NPls and interaction with other contaminants can potentiate the already reported toxic effects for each single compound through antagonistic or synergistic interactions [10,11,12,13].

Currently, the majority of ecotoxicological studies focus on exposures of one contaminant alone, underestimating the effects of contaminant mixtures that are occurring in soil due to anthropogenic activities. Few studies have been published on the effects on soil organisms due to co-contamination by MPls and other toxicants [14,15,16]. However, to the best of our knowledge, no study has been found regarding the impact of exposure to NPls together with other contaminants on soil fauna, except one in *Enchytraeus crypticus* where real plastic products were tested: a combination of plastics (acrylic, polyethylene, polypropylene and epoxy) containing NMs (copper oxide (CuO), iron(III) oxide (Fe_2_O_3_), organic pigment and multi-walled carbon nanotubes (MWCNT)) [17]. This is partly due to the limitations on the characterization of NPls in the soil [6] and, consequently, of the contaminant mixture [18].

In order to mitigate these knowledge gaps, the focus of the present study was the exposure of the commonly used standard terrestrial model invertebrate *E. crypticus* (oligochaeta) [19] to the combinations of polystyrene NPls and representatives of three major types of environmental contaminants: diphenhydramine (DPH, pharmaceutical), silver nitrate (AgNO_3_, metal) and vanadium nanoparticles (VNPs, nanomaterial). In prior studies, these contaminants have been shown to be toxic to different soil model organisms [10,20,21]. This understanding (about toxicological effects), together with the knowledge of their stability in soil (DPH) [21], widespread release (Ag) [20], chemical characteristics (V) such as oxidative state [10], and the observed long-term effects of NMs on soil systems [22] makes these compounds/materials relevant for co-contamination studies with NPls.

In the present study, it is hypothesized that in a co-contamination scenario, the interaction between NPls and each contaminant will result in a higher level of toxic effects, in particular in reproduction of *E. crypticus*, compared to the single exposures. This work provides novel information on the role of NPls in the hazardous potential of known contaminants to relevant soil model organisms such as enchytraeids, mimicking more realistic scenarios that already occur in the field, with potential long-term effects on ecosystem composition. Enchytraeids live in the litter layer and the upper mineral soil, feeding on fungal hyphae, microorganisms and dead organic matter [23]. Moreover, they greatly contribute to the acceleration of organic matter decomposition and nutrient recycling processes. Being a soft-bodied invertebrate, uptake is made via ingestion (e.g., food and soil particles) or via the body surface or dermis (used for gaseous exchange and water uptake) [24]. Previous studies already successfully used *E. crypticus* to test the effects of different anthropogenic contaminants (e.g., metals, NPls and NMs) [10,11,25].

## 2. Materials and Methods

### 2.1. Test Organism

*E. crypticus* (Enchytraeidae, Oligochaeta), Westheide and Graefe, 1992, was used for the tests. According to Directive 2010/63/EU of the European Parliament and of the Council of 22/9/2010, invertebrates such as *E. crypticus* are permitted biological models for scientific experimentation and are free from Ethical Statements. The cultures were kept in agar, consisting of Bacti-Agar medium (Oxoid, Agar No. 1) and a sterilized mixture of four salt solutions as previously described [10]. Cultures were fed on autoclaved ground oats twice per week.

### 2.2. Contaminant Test Characteristics 

Nanopolystyrene dispersion (10.06% solids in deionized water with 0.1% sodium dodecyl sulfate (SDS) and 0.05% sodium azide (NaN_3_)) was acquired from Bangs Laboratories, Inc. According to the supplier, NPls had mean diameter of 44 nm and a surface area of 1.299 × 10^14^ µm^2^/g. NPls dispersion was centrifuged prior to the ecotoxicity tests using a Vivaspin^®^ 2 mL ultrafiltration device (Bangs Laboratories, Inc., Fishers, IN, USA) to remove SDS and NaN_3_. Then, the NPls stock dispersion was characterized by hydrodynamic size (HS, Z-Average), evaluated by dynamic light scattering (Zetasizer Nano ZS, Malvern, OR, USA) and by zeta potential (ZP), assessed by electrophoretic light scattering (Zetasizer Nano ZS, Malvern) to verify the characteristics given by the supplier. The Zetasizer Nano ZS (Malvern) also allowed to obtain the polydispersity index (PdI) of the NPls dispersion. DPH hydrochloride (powder) was acquired from Sigma-Aldrich, with a molecular weight of 291.82 g/mol and a purity of 98% and an n-octanol/water partition coefficient (logKow) of 3.27. AgNO_3_ (powder) was acquired from PanReac, with a molecular weight of 169.87 g/mol and 99.8% purity. VNPs dispersion (2% in triton X-100 and water) was purchased from Nanoshel UK Limited (Cheshire, UK), presenting an average particle size (APS) between 80 and 100 nm and a purity of 99.9%. As for NPls, VNPs stock and test dispersions were characterized by HS and ZP.

### 2.3. Contaminant Interaction Characterization

To investigate the interaction between NPls and the other contaminants (DPH, AgNO_3_ and VNPs), HS, ZP and PdI of the test dispersions—NPls single exposures (1.5 and 300 mg/kg) combined with DPH, AgNO_3_ and VNPs (10 and 50 mg/kg)—were measured at day 0 of the ecotoxicity tests. 

### 2.4. Test Soil and Spiking Procedures

The natural standard LUFA 2.2 soil (Speyer, Germany) was used for the tests and had the following main characteristics: pH = 5.8, organic carbon = 1.71%, cation exchange capacity = 9.2 meq/100 g, maximum water-holding capacity (WHC) = 44.8% and grain size distribution of 8.9% clay, 13.9% silt and 77.2% sand.

The soil was dried (48 h; 60 °C) before use. The control soil was prepared by adding deionized water to adjust to the adequate moisture content (50% of the maximum WHC). Due to the presence of 2% of triton X-100 on the VNPs stock dispersion, a solvent control was also performed, adding the same volume of triton X-100 present in the highest tested concentration of VNPs (50 mg/Kg) (corresponding to 0.2% of triton X-100). For DPH and AgNO_3_ test solutions, they were dissolved in ultrapure water, no organic solvent needed. The required volumes of NPls test dispersions prepared in ultrapure water—single and combined with DPH, AgNO_3_ or VNPs—were added to the pre-moistened soil (in which water was added before) until 50% of the WHC maximum and mixed manually. Moreover, for the single exposures, test solutions of DPH and AgNO_3_ and test dispersions of VNPs, prepared in ultrapure water, were added to the soil, as described for NPls test dispersions. The replicates were mixed individually as recommended by the Organisation for Economic Cooperation and Development (OECD) guidelines [19]. Soil spiking was performed according to the following experimental conditions: single exposures of NPls (1.5 and 300 mg/kg) and DPH, AgNO_3_ and VNPs (10 and 50 mg/kg), as well as the binary mixtures of NPls with each co-contaminant (1.5 mg NPls/kg + 10 mg DPH/kg; 1.5 mg NPls/kg + 50 mg DPH/kg; 300 mg NPls/kg + 10 mg DPH/kg; 300 mg NPls/kg + 50 mg DPH/kg; 1.5 mg NPls/kg + 10 mg AgNO_3_/kg; 1.5 mg NPls/kg + 50 mg AgNO_3_/kg; 300 mg NPls/kg + 10 mg AgNO_3_/kg; 300 mg NPls/kg + 50 mg AgNO_3_/kg; 1.5 mg NPls/kg + 10 mg VNPs/kg; 1.5 mg NPls/kg + 50 mg VNPs/kg; 300 mg NPls/kg + 10 mg VNPs/kg; 300 mg NPls/kg + 50 mg VNPs/kg). The selected single VNPs, AgNO_3_ and NPls concentrations, the toxicities of which were previously studied [10,11,25], were used to examine the role of NPls in the toxicity of those contaminants. For DPH concentrations, its effects on *E. crypticus* were not known yet (single and combined with NPls). All the ecotoxicity tests started 1 day after soil spiking, similar to other previous published studies involving the testing of nano-based materials [22,26,27,28,29].

### 2.5. Enchytraeid Reproduction Test (ERT) Procedures

The ERT procedures followed the OECD guideline 220 [19], with adaptations. In short, 10 enchytraeids of synchronized age (17–19 days) were introduced in each test vessel, containing 20 g of moist soil and 11 mg of food (autoclaved ground oats). Starting tests with synchronized age, *E. crypticus* was an adaptation to the OECD guideline 220, allowing their development (i.e., maturation) in the contaminated media (i.e., test soil). Hence, the used time of exposure was 28 days (instead of 21 days). The test ran at 20 ± 1 °C and a 16 h: 8 h (light: dark) photoperiod. During the test, food (11 mg) and water content (based on weight loss) were replenished weekly. Four replicates per experimental condition (*n* = 4) were used. An additional replicate per condition (without organisms) was prepared to measure the pH values.

At the end of the test period, the organisms were fixed with ethanol and stained with 1% Bengal rose in ethanol (minimum of 4 hours). Soil samples were sieved through meshes with decreasing pore size (1.6, 0.5 and 0.3 mm) to separate the organisms from most of the soil and facilitate counting. Adult and juvenile enchytraeids were counted using a stereomicroscope, and survival (number of adults) and reproduction (number of juveniles) were evaluated.

### 2.6. Data Analysis

Graphics and statistical analysis were performed applying the Sigma Plot 14.0 software package (Munich, Germany). Shapiro–Wilk and Levene’s tests were applied to evaluate the normality and homoscedasticity of data, respectively. To assess differences between control and treatments, one-way analysis of variance (ANOVA) followed by Dunnett’s multiple comparison post hoc test were employed. When data did not follow a normal distribution, a non-parametric Kruskal–Wallis test was performed. Differences between control and solvent control were carried out using a Student’s *t*-test.

To assess the contribution of the interactions between NPls and the other contaminants (DPH, AgNO_3_ and VNPs) to the organisms’ survival and reproduction response, a two-way ANOVA was performed, followed by post hoc Dunnett’s test. 

Significant differences were accepted for a significance level of *p* < 0.05.

## 3. Results

### 3.1. Contaminant Interaction Characterization

NPls test dispersions (1.5 and 300 mg/kg) presented the expected HS with lower PdI (Table 1), being similar to the one measured at the stock dispersion (around 45 nm). The presence of DPH, for both tested concentrations, induced an increase in NPls HS and a less negative ZP value (Table 1). Similarly, in the test dispersions of NPls with VNPs, higher HS was detected, as well as a ZP closest to 0 (Table 1). The HS of VNPs stock and test dispersions (10 and 50 mg/kg) was around 90 nm and the ZP was −20 mV. The presence of AgNO_3_ did not induce any effect on NPls HS and ZP, once these values were similar when NPls were alone or combined with the metal (Table 1).

### 3.2. Enchytraeus crypticus Survival and Reproduction

**Figure 1:**
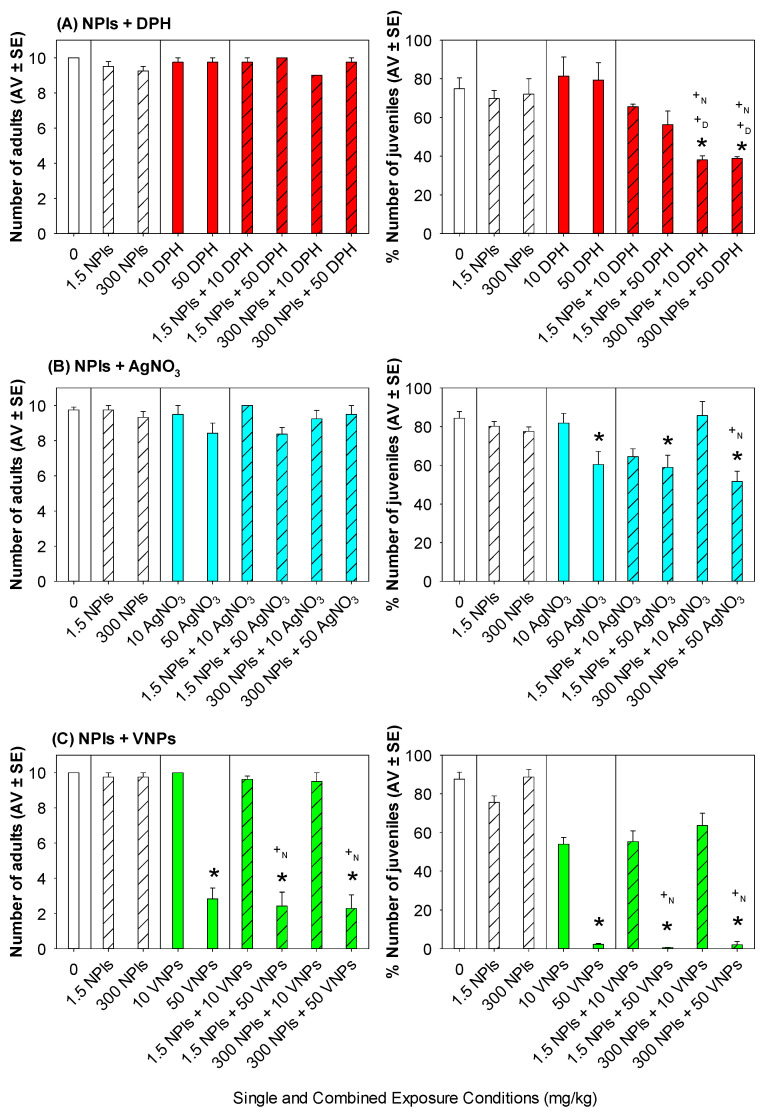
*E. crypticus* survival (number of adults) and reproduction (number of juveniles) after 28 days combined exposure in LUFA 2.2 soil to polystyrene nanoplastics (NPls) and (**A**) diphenhydramine (DPH), in red; (**B**) silver nitrate (AgNO_3_), in blue; or (**C**) vanadium nanoparticles (VNPs), in green. Dashed bars represent treatments with NPls. Data are expressed as average value (AV) ± standard error (SE). * Significant differences to control (*p* < 0.05). +D Significant differences to the correspondent DPH single exposure (*p* < 0.05). +N Significant differences to the correspondent NPls single exposure (*p* < 0.05).

The results of *E. crypticus* survival and reproduction after 28 days of exposure to soil contaminated with NPls, single and combined with DPH, AgNO_3_ or VNPs, are shown in Figure 1 and described in the following sections. The test validity criteria were fulfilled, as mortality in control was below 20%, the number of juveniles was above 25 and the respective coefficient variation was below 50%.

### 3.3. Enchytraeus crypticus Response to Contaminant Single Exposures 

After 28 days of exposure, no difference was observed in *E. crypticus* reproduction and survival between the control and 1.5 or 300 mg NPls/kg (*p* > 0.05, Figure 1). Similarly, no differences were observed between control and DPH single exposures (both 10 and 50 mg/kg; *p* > 0.05; Figure 1A).

AgNO_3_ decreased the organisms’ reproduction at the highest tested concentration (50 mg/kg; *p* < 0.05; Figure 1B), while exposure to 50 mg VNPs/kg caused a decrease in both reproduction and survival (*p* < 0.05; Figure 1C).

### 3.4. Enchytraeus crypticus Response to NPls and DPH Co-Contamination

After 28 days of exposure, the highest tested concentration of NPls (300 mg/kg) combined with 10 or 50 mg DPH/kg decreased the *E. crypticus* reproduction compared to control and the correspondent single exposures (*p* < 0.05; Figure 1A). From the two-way ANOVA, it was possible to identify a significant interaction between NPls and DPH in the reproduction response of *E. crypticus* (*p* < 0.05; Table 2).

### 3.5. Enchytraeus crypticus Response to NPsl and AgNO_3_ Co-Contamination

After 28 days of exposure, co-contamination of NPls (1.5 or 300 mg/kg) with the highest tested concentration of AgNO_3_ (50 mg/kg) decreased the *E. crypticus* reproduction, compared to control (*p* < 0.05; Figure 1B). In addition, significant differences in reproduction were observed for the combination 300 mg NPls/kg and 50 mg AgNO_3_/kg compared with the correspondent NPls single exposure (300 mg/kg; *p* < 0.05; Figure 1B). However, the effect of the combination (NPls + AgNO_3_) on the organisms’ reproduction was similar to the one observed in the correspondent single AgNO_3_ exposure (50 mg/kg; *p* > 0.05; Figure 1B). This finding was further confirmed by the results of the two-way ANOVA for both reproduction and survival with the presence of AgNO_3,_ showing a significant influence on the observed effect of the mixture (*p* < 0.05; Table 2), with no potential interaction between NPls and the metal occurring.

### 3.6. Enchytraeus crypticus Response to NPls and VNPs Co-Contamination

After 28 days of exposure, simultaneous exposure to NPls (1.5 or 300 mg/kg) and 50 mg VNPs/kg decreased the *E. crypticus* survival compared to control and the respective single NPls exposures (1.5 or 300 mg/kg; *p* < 0.05; Figure 1C), with no differences to the correspondent single VNPs exposure (50 mg/kg; *p* > 0.05; Figure 1C). Organisms’ reproduction also decreased for the combination NPls (1.5 or 300 mg/kg) and 50 mg VNPs/kg (*p* < 0.05; Figure 1C), compared to control and the respective single NPls exposures (1.5 or 300 mg/kg; *p* < 0.05; Figure 1C), with no differences to the correspondent single VNPs exposure (50 mg/kg; *p* > 0.05; Figure 1C). No significant interaction between NPls and VNPs was found by the two-way ANOVA (*p* < 0.05; Table 2).

## 4. Discussion

### 4.1. Enchytraeus crypticus Response to Contaminant Single Exposures 

Despite the few studies available involving *E. crypticus* and polystyrene-based NPls [11,30], one study showed that NPls (mean diameter: 60 nm) induced no effects in organisms’ survival or reproduction at concentrations from 0.015 to 900 mg/kg [11]. The previous study is in accordance with the findings of the current study (absence of effects of NPls). However, exposure to 10% (*w*/*v*) NPls (0.05–0.1 μm particle size) resulted in a significant decrease in *E. crypticus* cocoon production after 7 days [30].

Currently, there is no information on the effects of DPH in survival and reproduction of soil organisms, and limited information on aquatic organisms [31,32]. Acute exposure to 2 mg/L DPH induced mortality in *Ceriodaphnia dubia* [32], while *Daphnia magna* reproduction was affected at concentrations above 0.8 µg/L [31]. The present study showed that DPH (10 and 50 mg/kg) induced no effects in the survival and reproduction of *E. crypticus* after 28 days. However, as DPH is considered persistent in soil [21] and can be degraded, due to interactions with natural organic matter [33] and existing microbial structure [34], it can release compounds that may lead to toxicity in long-term exposure. 

In the present study, the decrease in organisms’ reproduction observed after the exposure to 50 mg AgNO_3_/kg was expected. The tested concentration is within the confidence interval of the 10% effect concentration (EC_10_) estimated by Gomes et al. [35] and between the 20% EC (EC_20_) and 50% EC (EC_50_) estimated by Bicho et al. [25] for *E. crypticus*. As no effects were observed for *E. crypticus* survival, it is possible that AgNO_3_ may delay the hatching process and embryotoxicity via blocking of Ca channels, as previously proposed [25,36].

Prior studies on the effects of VNPs on biological systems are in limited number [10,37,38]. However, vanadium dioxide (VO_2_) NPs have been shown to produce effects at the minimal tested concentrations (2.5 µg/mL) in human lung cells as well as Gram positive bacteria, related to an increased production of reactive oxygen species (ROS) [37,38]. More recently, VNPs have been shown to be toxic to soil invertebrates, namely affecting *E. crypticus* reproduction (28 days EC_50_ = 11.0 ± 1.5 mg/kg) as well as survival [10]. The findings of the current study corroborate the previous work, as exposure to 50 mg VNPs/kg induced a decrease in organisms’ reproduction and survival.

### 4.2. Enchytraeus crypticus Response to NPl and DPH Co-Contamination

Co-contamination of NPls and DPH resulted in a higher degree of effect in *E. crypticus* reproduction when compared with the single exposures (where no effect was observed). Furthermore, the significant interaction observed for NPls and DPH (synergism) indicates that there may be a combined effect of the mixture, namely a potentiation by NPls to DPH toxicity [39]. Similar potentiation has been observed in soil microbial communities after the exposure to the combination NPls and platinum-based drugs [40]. Concerning aquatic organisms, zebrafish survival was affected by the co-exposure of NPls and the pharmaceutical simvastatin [41]. In fish cell lines, the effects on cell viability of the combination polystyrene NPls and pharmaceuticals were dependent on the cell line used and the tested pharmaceutical [42]. Prior studies show that polystyrene NPls can adsorb other organic compounds, such as the antibiotic ciprofloxacin [43], suggesting that NPls could act as carrier for DPH. On the other hand, it is described that plastic particles can interfere with soil structure and properties, such as the natural organic matter [44,45], which can alter the (potential) toxicity of the contaminants to the organisms. The possible interaction between NPls and DPH can be supported by the NPls characterization results. The combination with DPH, regardless of the concentration, leads to increased HS and ZP values of NPls, suggesting aggregation/agglomeration processes and/or a potential adsorption/binding between NPls and DPH.

In addition, the nature of the pharmaceutical is relevant for the adsorption process, as it is mediated mainly by hydrophobic, Van der Waals and hydrogen bonding, together with the role of environmental pH, as presented by McDougall and prior studies [46]. In the present study, soil pH was neutral, lowering the desorption potential, while the DPH logKow was higher than 2.4, promoting the adsorption of the pharmaceutical by NPls through hydrophobic interactions and consequently the observed effects [46].

### 4.3. Enchytraeus crypticus Response to NPls and AgNO_3_ Co-Contamination

In the combined exposure NPls and AgNO_3_, it was found that the predominant driver in the observed toxicity was the presence of AgNO_3_. No different effects were observed between AgNO_3_ single and combined exposures, suggesting no interaction effect between the metal and NPls. This can be explained by the absence of a functional group (such as carboxyl (COOH) or amino (NH_2_)) in the NPls used (not functionalized). If the tested NPls contain a functional group, it could promote the binding of the Ag ions with the NPls, and, consequently, form stable complexes. This complex (NPls + AgNO_3_) could cause a different toxicity than expected. Indeed, a previous study with the brine shrimp *Artemia franciscana* involving two types of metal-based salts (potassium dichromate (K_2_Cr_2_O_7_) and copper sulfate (CuSO_4_)) co-exposed with amino-functionalized polystyrene NPls, found an interaction effect between the metals and NPls [47]. In the co-exposure of wheat (*Triticum aestivum L.*) to polystyrene NPls and cadmium (Cd), the presence of polystyrene induced a slight alleviation of Cd-induced toxicity [48]. In addition, for the aquatic species *A. franciscana*, polystyrene NPls decreased the immobilization rate induced by the single CuSO_4_ exposure [47]. On the other hand, in the terrestrial environment, *Eisenia fetida* exposure to NPls in arsenic (As) and Cd contaminated soil promoted antioxidant response (glutathione S-transferase (GST) activity and malondialdehyde (MDA) content increased) [49]. In the aquatic environment, the presence of gold (Au) exacerbated the toxic effects of polystyrene NPls in zebrafish embryos [50], and the co-contamination of polystyrene NPls with K_2_Cr_2_O_7_ induced a higher immobilization rate in *A. franciscana* larvae [47]. The described results indicate that the effects resulting from the combination of NPls and metals can depend on, among others, metal type, tested concentrations and studied species. The no interaction effect found for NPls and AgNO_3_ can be supported by the results from the characterization of NPls, since the HS and ZP NPls values maintained unaltered in the presence of AgNO_3_. This is likely a result of the dissolution of AgNO_3_, decreasing the potential for interactions with less hydrophilic NPls.

### 4.4. Enchytraeus crypticus Response to NPls and VNPs Co-Contamination

It was clear that VNPs were the major driver of toxicity, as the combination of NPls with VNPs did not inhibit or increase the VNPs toxicity found in the single exposures. Similar to other metal-based NPs (such as Ag), VNPs can release V ions and cause toxicity [25]. It is possible that, due to their similar sizes (nano), the agglomeration/aggregation processes occurring between VNPs and NPls may promote their uptake and increase the potential for ion release within the organisms [51]. Indeed, the characterization of the test dispersions of NPls with VNPs showed increased HS and ZP typical of agglomeration/aggregation processes, when compared with the characterization of each individually. For silver nanoparticles (AgNPs) and polystyrene NPls co-exposure to *Chlamydomonas reinhardtii*, there was no increased uptake of plastic but of silver, promoting toxicity [52]. As such, the mechanisms by which the joint exposure affected NPs-related toxicity should be further looked upon, with the use of sub-lethal concentrations, widening the concentration range tested, as well as looking at other endpoints, such as oxidative stress biomarkers, cocoon production and the metal accumulation in tissues.

### 4.5. Comparison between Combined Exposures and Challenges

The use of three contaminants with distinct nature (organic, metal ionic form and metal nanoparticulate form) in combination with NPls showed clear differences in terms of impact in *E. crypticus* response. The combination which caused the most effects was VNPs and NPls, which caused effects for both tested endpoints: survival and reproduction. However, these effects were due to the VNPs exposure, independent of the presence of NPls. Comparing the results of combined exposures with the correspondent single exposures, different interaction effects, including no interaction and synergism, were found. The presence of NPls did not have an interference (showing no interaction) on the observed effects of VNPs and AgNO_3_. Yet, the combination of DPH and NPls resulted in toxic effects that were not observed in the single exposures, i.e., there was an interaction effect related to the synergy between both contaminants.

The complexity of the exposure matrix (soil) provides several challenges to assessing how NPls interact with the surrounding environment and other contaminants [6]. The presence of DPH in the mixture with NPls seemed to promote agglomeration/aggregation processes (supported by the contaminant interaction characterization results) in a concentration-dependent manner, showing the potential synergistic effects of the mixture. Moreover, a possible absorption or linking of NPls with DPH may also be explained in the increased HS and ZP values of NPls and the consequent interaction effects between the contaminants (potentiation). Per comparison with DPH, it seemed that the presence of VNPs induced a similar effect on the NPls physical-chemical parameters (increased HS and ZP values, typical of agglomeration/aggregation processes). However, there was no proof of significant interaction between NPls and VNPs that supported the toxic effects found in organisms’ reproduction after the exposure to the mixture NPls and VNPs. In fact, in the single exposures, no effects were detected for DPH, whereas for VNPs, toxic effects were found for both single and combined exposures with NPls. Additionally, for NPls + VNPs test dispersions, the obtained characterization results were from both nano forms (NPls and VNPs). Therefore, no direct comparison should be made with the results found for the mixture NPls + DPH test dispersions, where the values obtained only referred to NPls. 

The obtained data revealed that the nature of the co-contaminant (e.g., organic vs. inorganic) had a key role in the toxicity mediated by NPls. In recent studies with organic pollutants and MPls in soil it has been shown that the latter can act as adsorbents for hydrophobic organic compounds (HOC), reducing its availability and decreasing the toxicity potential [53,54]. However, the current study showed otherwise. As such, the specificity of functional groups may be determinant for the interaction between MPls/NPls and co-contaminants [55], which in turn can modulate their toxicity, similar to what occurs with surface modifications of NMs in soil multispecies systems (SMS) [56]. On the other hand, NPls masking the contaminant, even at low concentrations, can increase its potential internalization in organisms, similar to what occurred with NPls and simvastatin [41] or ciprofloxacin [43]. This can explain the higher toxicity of NPls + DPH compared with the single exposures (no effect). Considering the role of NPls in the toxicity of the tested contaminants, the results indicated a higher hazardous potential for the combination of the pharmaceutical and NPls compared with the other tested combinations (NPls with metal or NMs). Further studies on these types of interactions, up to the formation of coronas between organic species and NPls in the soil matrix [57], should be key towards the understanding of the mechanisms for NPl joint effects.

## 5. Conclusions

The present study provided key information on the potential hazard to terrestrial organisms of soil co-contaminated with NPls and other contaminants, specifically pharmaceuticals (DPH), metals (AgNO_3_) and NMs (VNPs). Effects were observed for both single and combined exposures of VNPs (reduction of organisms’ survival and reproduction) and AgNO_3_ (reduction of organisms’ reproduction), in a concentration-related manner. Since these negative effects were similar in single and combined exposures, it showed that no relevant interaction occurred between NPls and VNPs or AgNO_3_. For these combinations, the presence of NPls did not limit the binary mixture effect. However, considering the combination of NPls with the pharmaceutical, a higher toxicity of NPls + DPH (reproduction decreased) was found compared with the single exposures (no effect). In this case, a significant interaction was observed for NPls and DPH (synergism). The present results showed that the presence of NPls should not be ignored when performing environmental risk assessment of other contaminants. Due to the demonstrated role of NPls as a vector for other contaminants, further studies are encouraged to a more complete understanding about the NPls interactions and mechanisms of toxicity involved when NPls are in complex mixtures of contaminants. One key aspect that should be studied is the observation and characterization of molecular interactions between NPls and other components, such as natural organic matter in complex environmental matrices such as soil, or other pollutant matrices such as wastewater sludge, which require the combination of state-of-the-art techniques. These advances, coupled with the evidence presented in the current paper, will provide important information to fill the knowledge gap on the hazardous potential of NPls to the environment.

## Figures and Tables

**Table 1:** Characterization of the test dispersions: single exposures of polystyrene nanoplastics (NPls) (1.5 and 300 mg/kg) and their combinations with diphenhydramine (DPH), silver nitrate (AgNO3) and vanadium nanoparticles (VNPs). Z-average—Hydrodynamic diameter; PdI—polydispersity index.

Contaminants (mg/L)	Z-Average (d.nm)	PdI	Zeta Potential (mV)
1.5 NPls	45.1	0.1	−26.8
1.5 NPls + 10 DPH	281.5	0.6	−19.2
1.5 NPls + 50 DPH	291.5	0.7	−16.9
1.5 NPls + 10 AgNO_3_	44.4	0.1	−32.2
1.5 NPls + 50 AgNO_3_	46.7	0.1	−32.6
1.5 NPls + 10 VNPs	194.3	0.4	−5.2
1.5 NPls + 50 VNPs	211.1	0.5	−13.3
300 NPls	44.5	0.1	−26.7
300 NPls + 10 DPH	294.4	0.4	−20.0
300 NPls + 50 DPH	324.3	0.5	−19.3
300 NPls + 10 AgNO_3_	47.1	0.2	−33.7
300 NPls + 50 AgNO_3_	47.2	0.2	−37.4
300 NPls + 10 VNPs	386.5	0.5	−14.0
300 NPls + 50 VNPs	324.3	0.6	−15.9

**Table 2:** Two-way ANOVA results for *E. crypticus* survival and reproduction after 28 days combined exposure to polystyrene nanoplastics (NPls) and diphenhydramine (DPH), silver nitrate (AgNO_3_) or vanadium nanoparticles (VNPs) in LUFA 2.2 soil. Significant interactions (*p* < 0.05) are highlighted in bold.

Survival							Reproduction						
Source of Variation	DF	SS	MS	F	P	α	Source of Variation	DF	SS	MS	F	P	α
NPls	2	1.722	0.861	4.895	0.015	0.653	NPls	2	4526.261	2263.130	17.579	<0.001	0.999
DPH	2	0.722	0.361	2.053	0.148	0.215	DPH	2	1160.891	580.445	4.509	0.022	0.598
NPls × DPH	4	1.111	0.278	1.579	0.208	0.168	NPls × DPH	4	2007.759	501.940	3.899	0.014	0.693
Residual	27	4.750	0.176				Residual	24	3089.710	128.738			
Total	35	8.306	0.237				Total	32	11,102.534	346.954			
NPls	2	0.211	0.106	0.117	0.890	0.05	NPls	2	429.692	214.846	1.274	0.293	0.0898
AgNO_3_	2	7.029	3.515	3.910	0.029	0.534	AgNO_3_	2	4508.968	2254.484	13.373	<0.001	0.993
NPls × AgNO_3_	4	5.230	1.308	1.455	0.236	0.145	NPls × AgNO_3_	4	898.744	224.686	1.333	0.278	0.116
Residual	37	33.256	0.899				Residual	34	5731.830	168.583			
Total	45	49.826	1.107				Total	42	11,865.789	282.519			
NPls	2	1.656	0.828	0.521	0.598	0.05	NPls	2	437.659	218.829	3.871	0.028	0.534
VNPs	2	619.438	309.719	194.845	<0.001	1	VNPs	2	67,047.587	33,523.793	593.022	<0.001	1
NPls × VNPs	4	0.143	0.0357	0.0225	0.999	0.05	NPls × VNPs	4	382.579	95.645	1.692	0.169	0.207
Residual	43	68.351	1.590				Residual	44	2487.341	56.530			
Total	51	722.981	14.176				Total	52	74,624.249	1435.082			

**DF**—degrees of freedom; **SS**—sum of squares; **MS**—mean squares; **F**—F ratio; **P**—*p* values; **α**—power of the performed test.

## Data Availability

The data are available on request from the corresponding author.
